# Excess Mortality, Causes of Death and Life Expectancy in 270,770 Patients with Recent Onset of Mental Disorders in Denmark, Finland and Sweden

**DOI:** 10.1371/journal.pone.0055176

**Published:** 2013-01-25

**Authors:** Merete Nordentoft, Kristian Wahlbeck, Jonas Hällgren, Jeanette Westman, Urban Ösby, Hassan Alinaghizadeh, Mika Gissler, Thomas Munk Laursen

**Affiliations:** 1 Psychiatric Centre Copenhagen, University of Copenhagen, Faculty of Health Sciences, Copenhagen, Denmark; 2 Nordic Research Academy in Mental Health, Nordic School of Public Health, Gothenburg, Sweden; 3 Department of Medical Epidemiology and Biostatistics, Karolinska Institutet, Huddinge, Stockholm, Sweden; 4 Department of Neurobiology, Care Sciences and Society, Centre for Family Medicine, Karolinska Institutet, Huddinge, Stockholm, Sweden; 5 Department of Molecular Medicine and Surgery, Neurogenetics Unit, Center for Molecular Medicine, Karolinska Institutet, Stockholm, Sweden; 6 THL National Institute for Health and Welfare, Helsinki, Finland; 7 National Centre for Register-Based Research, Aarhus University, Aarhus, Denmark; University of Queensland, Australia

## Abstract

**Background:**

Excess mortality among patients with severe mental disorders has not previously been investigated in detail in large complete national populations.

**Objective:**

To investigate the excess mortality in different diagnostic categories due to suicide and other external causes of death, and due to specific causes in connection with diseases and medical conditions.

**Methods:**

In longitudinal national psychiatric case registers from Denmark, Finland, and Sweden, a cohort of 270,770 recent-onset patients, who at least once during the period 2000 to 2006 were admitted due to a psychiatric disorder, were followed until death or the end of 2006. They were followed for 912,279 person years, and 28,088 deaths were analyzed. Life expectancy and standardized cause-specific mortality rates were estimated in each diagnostic group in all three countries.

**Results:**

The life expectancy was generally approximately 15 years shorter for women and 20 years shorter for men, compared to the general population. Mortality due to diseases and medical conditions was increased two- to three-fold, while excess mortality from external causes ranged from three- to 77-fold. Mortality due to diseases and medical conditions was generally lowest in patients with affective disorders and highest in patients with substance abuse and personality disorders, while mortality due to suicide was highest in patients with affective disorders and personality disorders, and mortality due to other external causes was highest in patients with substance abuse.

**Conclusions:**

These alarming figures call for action in order to prevent the high mortality.

## Introduction

Excess mortality is well-known among patients with mental disorders compared to the general population[1]–[8]. The excess mortality is not solely explained by increased risk of suicide and other external causes of death; it is also due to diseases and medical conditions. A recent Danish study confirms that mortality from cardiovascular disease, malignant neoplasms, respiratory diseases and endocrine and metabolic conditions was more than twofold higher for both men and women with schizophrenia and affective disorders compared to the general population. [6].

The need for more detailed analyses of the excess mortality is underlined by the fact that some recent studies indicate that the overall mortality gap[9]–[12] or the mortality gap due to cardiovascular diseases [13] increased over time. However, in a large register-based study for Denmark, Finland and Sweden, we could not confirm this finding. [14].

Only few studies investigated mortality for total national samples, [3], [10], [12] and hitherto, no studies have analyzed in detail mortality in different diagnostic groups for total national populations in several countries. In Denmark, Finland and Sweden, unique possibilities exist to investigate mortality figures for national samples of patients with mental disorders, due to their mandatory national longitudinal registers containing information about treatment for mental disorders, deaths and their causes. A Nordic research collaboration to exploit these register-based possibilities has shown that this mortality decreased slightly from 1987 to 2006, and also that it still is at a very high level. Patients with severe mental disorders have a life expectancy that is approximately 15–20 years shorter than the general population in Denmark, Finland and Sweden. [14] The excess mortality of people with severe mental disorders constitutes a serious public health problem. To enhance possibilities for mortality prevention, it is necessary to understand the excess mortality in detail, i.e. in different diagnostic groups by main causes of death.

Register-based studies of schizophrenia have previously shown that the first year of treatment is associated with the highest risk of suicide.[15]–[18] To our knowledge, mortality due to other causes of death has not previously been investigated in different diagnostic groups in a sample of recent-onset cases, and it is therefore relevant to compare the risk of suicide and other causes of death during the different phases in the first years of treatment.

The purpose of the present study is to investigate life expectancy and mortality due to specific causes of death in different diagnostic groups as well as to investigate excess mortality in the first year after first admission in comparison with subsequent years.

## Methods

In each of the three countries, we received permission from the data-maintenance agencies to access the register data.

### Ethics

The study was approved by ethical committees and data protection agencies in each of the three countries. According to the legislation in the three countries, no informed consent from participants was needed because data were analyzed anonymously.

The study was approved by the Danish Data Protection Agency (2000-41-0307).

The permission to create Finnish research database was given by STAKES (National Research and Development Centre for Welfare and Health, currently THL National Institute for Health and Welfare), Statistics Finland and National Social Insurance Institution. The Data Protection Ombudsman (Tietosuojavaltuutettu) gave his statement before the study data was created, as requested by the national legislation on data protection.

We have received ethical permission for this project from the ethics committee in Gothenburg, Sweden (Dnr 130-08).

### Risk Population

The risk population was identified from discharge diagnoses recorded in the nationwide population-based hospital discharge registers. In these registers, the Nordic countries use the International Classification of Diseases (ICD), established by the World Health Organization (WHO), for defining and classifying psychiatric and physical diseases. During the period 1987–2006, three ICD versions were used in Nordic hospital discharge and cause-of-death registers. We used the main diagnoses recorded for each hospitalization, and categorized them according to ICD-10 diagnose groups (WHO, 1992). The diagnoses in ICD-8 and ICD-9 were transformed to ICD-10 diagnoses.

A cohort of all patients with a primary diagnosis of any schizophrenia spectrum disorder, affective disorder, substance abuse or personality disorder (ICD-10: F10–F39 and F60–F69) was retrieved from the hospital registers.

Patients with a diagnosis of intellectual disability (F70–79 and equivalent diagnoses in ICD-8 and ICD-9) at any point in time were excluded. Hospitalizations due to organic mental disorders, e.g. dementia (F00–09), resulted in the subject’s exclusion, starting from the first hospitalization due to dementia and any episode thereafter. Patients with a diagnosis of intellectual disability and dementia were excluded, because of the high risk of pre-mature mortality inherent in the organic nature of these conditions.

In order to form a recent cohort of incident cases, all patients admitted to a psychiatric department or a somatic department with one of the above-mentioned primary diagnoses sometime during the period 1 January 2000 to 31 December 2006 were included. We were able to ensure that the cohort was a recent-onset cohort by establishing a wash-out period that excluded patients admitted with a main diagnosis with one of the above-mentioned diagnostic codes in ICD-10, or the corresponding figures in ICD-8 and ICD-9, during the period between 1 January 1987 and 31 December 1999. Thus, the risk population consists of patients who had not been hospitalized during the previous 13 years for any of the disorders being studied. By using this method of selection, we aimed to include a risk population of incident cases. Patients were followed until death or 31 December 2006, whichever came first. Life expectancy and standardized cause-specific mortality rates were calculated for each of the diagnostic groups in each of the three countries.

By the end of 2006, the total population in Denmark, Finland and Sweden was 19.8 million. The cohorts in the three study countries consisted of 270,770 recent-onset patients (145,799 men and 124,971 women). They were followed for 912,279 person years, and 28,088 deaths and their causes were analyzed.

### Psychiatric Diagnoses

The patient groups were divided into diagnostic groups according to their main discharge diagnosis from ICD-10 (or corresponding figures in ICD-8 and ICD-9). Patients should have been diagnosed at least once with one of the following conditions, listed below:

1. Patients who at least once had a diagnosis in schizophrenia spectrum (ICD-10 F20–F29, ICD-9: 295, 297, 298B, 298C, 298E, 298W, 298X, 301C, ICD-8: 295, 297, 298.29, 298.39, 298.89, 298.99, 299.05, 299.09, 301.09, 301.29 (DSM IV: 295.10–295.90, 297.1, 297.3, 298.8–298.9, 301.22)).
2. Affective disorders (ICD-10 F30–F39, ICD-9: 296, 311, 298A, 300E, 301B, and ICD-8: 296, 298.09, 298.19, 300.49, 301.19 (DSM IV: 296.00–296.89, 300.4, 301.13, 311)).
3. Substance abuse (ICD-10: F10–F19, ICD-9: 291, 292, 303, 304, 305 and ICD-8: 291, 303, 304 (DSM IV: 305.00, 303.90, 291.0–291.9, 303.00, 305.20–305.90, 304.20–304.90, 292.0–292.9)).
4. Personality disorders (ICD-10: F60–F69, ICD-9: 301A, 301D, 301E, 301F, 301G, 301H,301J, 301W, 301X, 312, and ICD-8: 300.19, 301.49, 301.59, 301.69, 301.79, 301.80, 301,81, 301.82, 301.83, 301,84 (DSM IV: 301.0–301.9)).

### Causes of Death

Information on causes of death is collected in national cause-of-death registers. The main cause-of-death is based on the information available in the death certificate. The causes of death are determined either by the treating physician or by the forensic examiner (for unclear deaths or deaths from injuries and accidents including suicides) in each study country. There are no international specific guidelines for the forensic examiners, but WHO has a detailed instructions and recommendations for registration, coding and classification of causes of death.

For this study, we analyzed separately deaths from diseases and medical conditions (ICD-10: A00–R99), suicides (ICD-10: X60-84), and other external causes than suicides (ICD-10: V01–Y98, excluding X60-84). The deaths due to diseases and medical conditions were divided into: infections (ICD-10:A00–B99, G00–G09, J00–J22, K35–K37), malignant neoplasms (ICD-10: C00–D09), diseases of the circulatory system (ICD-10: I00–I99), diabetes (ICD-10: E10–E14), and other causes (comprising the rest of the causes).

### Nordic Registers

#### Denmark

The Danish Psychiatric Central Register [19] covers all psychiatric inpatient facilities in Denmark and has been computerized since 1969. The diagnostic system used until 1993 was ICD-8 (World Health Organization 1971) and ICD-10 (World Health Organization 1994) since 1994.

The Danish Cause of Death Register contains computerized information about all deaths of Danish citizens and residents, date of death, way of dying and causes of death. The register has a high level of completeness and its validity has been evaluated with excellent results. [20].

#### Finland

The Finnish Hospital Discharge Register includes data on all inpatient episodes on an individual level since 1969. For classification of diagnoses, ICD-8 was used during the period 1969–1986, ICD-9 with Diagnostic and Statistical Manual, 3^rd^ revised edition (DSM-III-R) criteria in 1987–1995 and ICD-10 since 1996. The register has been found to be a valid and reliable tool for epidemiological research. [21].

The Finnish Cause of Death Register records data on the deaths of all citizens and permanent residents in Finland. The register has a high level of completeness. All diagnoses of the causes of death have to pass a routine validation, carried out by regional medical officers and physicians at Statistics Finland. Generally, the quality has been found to be excellent. [22].

#### Sweden

The Swedish Hospital Discharge Register covers all hospitals in all regions in Sweden since 1987. It contains data on all hospital admissions and discharge diagnoses according to ICD. For diagnosis, ICD-9 was used in 1987–1996 and ICD-10 from 1997 onwards. The register has a high level of completeness. [23].

The Swedish Cause of Death Register contains information about deaths of all citizens and permanent residents in, classified according to ICD-9 and ICD-10. The under-reporting is low (less than 0.4% in 1996).

### Statistical Analysis

The risk population consisted of a cohort of all patients admitted at least once during the period 1 January 2000 to 31 December 2006. Mortality figures were based on deaths during the same period.

We used the Nordic standard population for year 2000 for standardizing mortality rates. [24] The standardized mortality rates among mental health patients were compared to general population mortality rates (WHO 2009), calculating the observed/expected ratio. (WHO: Mortality indicators by 67 causes of death, age and sex (HFA-MDB). World Health Organization, Regional Office for Europe. Copenhagen 2010. (http://data.euro.who.int/hfamdb/)).

For patients hospitalized for specific mental disorders, we calculated life expectancy at 15 years, separately for men and women, by using standard life-table method with one-year age stratification. Life expectancy at a given age represents the average number of years of life remaining if a group of persons at that age were to experience the mortality rates for a particular year over the course of their remaining life. Life expectancy at birth is a summary measure of the age-specific all-cause mortality rates in an area in a given period. [25] We used published WHO life expectancy data for general population comparisons. For each specific cause of death, we calculated cause-specific standardized mortality rates. Cause-specific standardized mortality rates (divided into deaths from external causes, and from diseases and medical conditions) were analyzed for the period of time since the first discharge from inpatient care, divided in first year deaths versus deaths after the first year since first hospital discharge.

## Results

**Figure 1:**
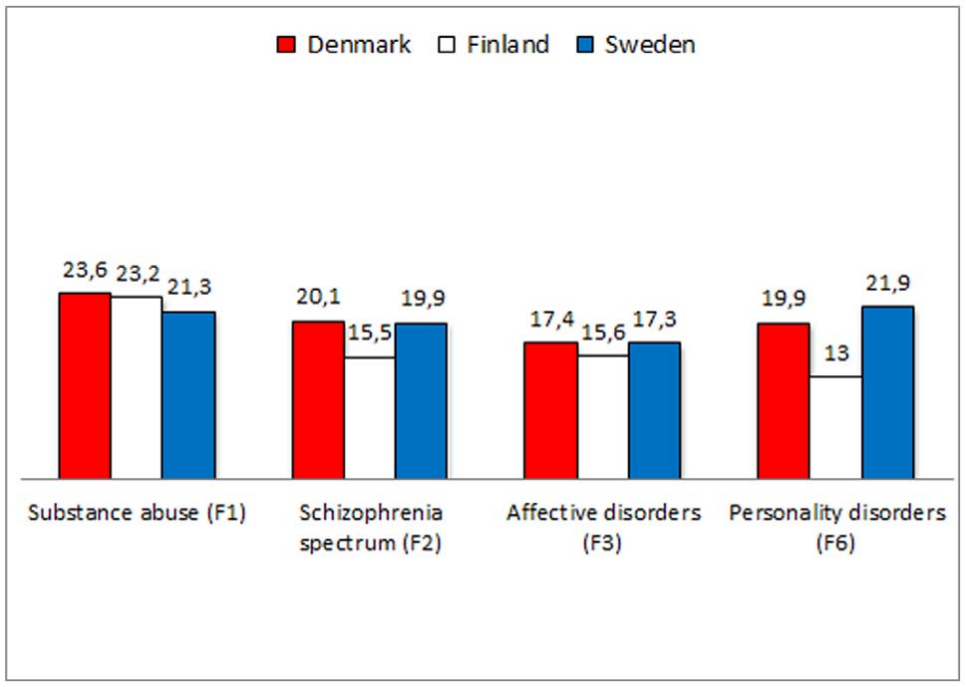
Difference in life expectancy among 145,799 men with recent onset mental illness in Denmark, Finland and Sweden compared to the general population.

**Figure 2:**
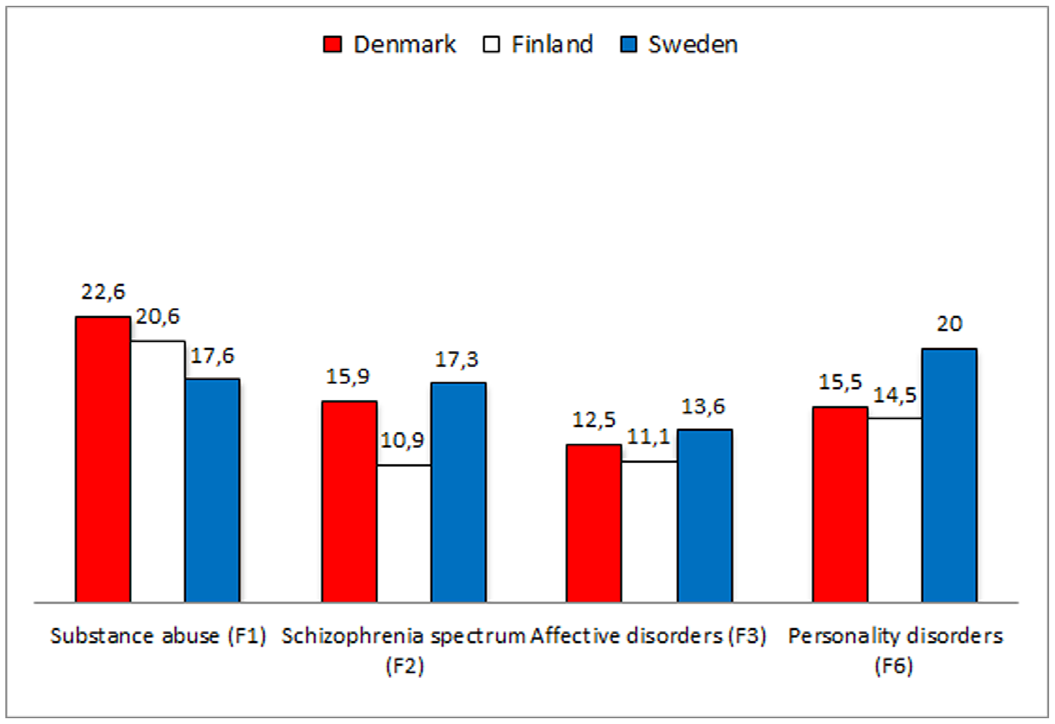
Difference in life expectancy among 124,971 women with recent onset mental illness in Denmark, Finland and Sweden compared to the general population.

Life expectancy was shortened for both men and women in all diagnostic groups. Generally, substance abuse disorders were associated with the shortest life expectancy – less so in Sweden than in Denmark and Finland – and affective disorders with the longest (tables 1 and 2, figures 1 and 2). All patients with all mental disorders, however, had a reduced life expectancy of at least ten years. For men with psychiatric disorder, the difference in life expectancy ranged between 13.0 and 23.6 years; the figures for women ranged from 11.1 to 22.6 years. The number of lost life years was highest for patients in Denmark in all diagnostic categories for both men and women.

**Table 1:** Life expectancy and specific causes of death for substance-related disorders, schizophrenia-like psychoses, affective disorders and personality disorders in men, 2000–2006 in Denmark, Finland and Sweden.

	Substanceabuse (F1)	Schizophreniaspectrum (F2)	Affectivedisorders (F3)	Personalitydisorders (F6)#
**Total risk population**				
Denmark	22,998	5,830	9,442	2,032
Finland	24,404	7,953	13,931	1,402
Sweden	33,376	6,014	16,765	1,652
**Person years**				
Denmark	71,280	18,384	27,911	6,647
Finland	81,510	29,433	50,005	5,832
Sweden	108,690	20,686	50,437	5,257
**Age groups**				
Denmark <30 years	8,867	3,069	2,130	1,197
Denmark 30 years and older	14,131	2,761	7,312	835
Finland <30 years	3,898	2,856	3,462	722
Finland 30 years and older	20,506	5,097	10,469	680
Sweden <30 years	12,175	2,143	3,457	731
Sweden 30 years and older	21,201	3,871	13,308	921
**Deaths**				
Denmark	2,755	443	1,252	123
Finland	3,975	834	1,465	99
Sweden	2,931	584	2,073	106
**Standardized O/E**				
Denmark	3.5	3.3	2.6	3.7
Finland	3.8	2.9	2.5	3.4
Sweden	2.9	3.3	2.5	3.8
**O/E Circulatory system**				
Denmark	2.0	2.4	2.0	-
Finland	2.8	2.5	2.0	-
Sweden	2.1	2.9	1.8	-
**O/E Cancer**				
Denmark	1.7	2.0	1.8	-
Finland	1.6	1.6	1.4	-
Sweden	1.6	2.0	1.5	-
**O/E Infections**				
Denmark	3.5	4.7	3.3	-
Finland	2.7	5.0	2.5	-
Sweden	2.6	4.5	2.3	-
**O/E Diabetes**				
Denmark	3.3	1.7	1.9	-
Finland	1.7	0.5	2.0	-
Sweden	2.4	2.3	2.6	-
**O/E Other diseases**				
Denmark	7.0	4.5	2.8	-
Finland	5.7	3.7	2.5	-
Sweden	4.1	3.7	2.3	-
**O/E All diseases and medical conditions**				
Denmark	3.3	2.9	2.2	3.3
Finland	3.1	2.6	2.0	3.2
Sweden	2.4	2.9	1.8	3.4
**O/E Suicides**				
Denmark	12.3	23.0	36.6	29.5
Finland	9.4	12.5	18.3	10.7
Sweden	13.9	22.7	35.6	23.7
**O/E Other external causes**				
Denmark	7.9	6.5	3.8	5.5
Finland	9.6	3.0	2.9	4.0
Sweden	9.3	5.6	3.5	6.4
**Life expectancy in risk population. Years**				
Denmark	52.1	55.6	58.3	55.8
Finland	52.4	60.1	60.6	62.0
Sweden	56.9	58.3	60.9	56,3
**Life expectancy in general population. Years**				
Denmark	75.7	75.7	75.7	75.7
Finland	75.6	75.6	75.6	75.6
Sweden	78.2	78.2	78.2	78.2
**Difference in LE. Years**				
Denmark	23.6	20.1	17.4	19.9
Finland	23.2	15.5	15.6	13.0
Sweden	21.3	19.9	17.3	21.9

# The number of cases with personality disorders did not allow detailed analyses of specific cause of death from different medical disorders.

**Table 2:** Life expectancy and specific causes of death for substance-related disorders, schizophrenia-like psychoses, affective disorders and personality disorders in women, 2000–2006 in Denmark, Finland and Sweden.

	Substanceabuse (F1)	Schizophreniaspectrum (F2)	Affectivedisorders (F3)	Personalitydisorders (F6)#
**Total risk population**				
Denmark	11,422	5,317	15,324	3,649
Finland	7,590	9,285	23,077	1,368
Sweden	20,381	6,571	25,503	3,074
**Person years**				
Denmark	35,807	16,572	46,820	12,095
Finland	27,061	32,998	82,170	5,359
Sweden	67,088	20,686	80,237	9,314
**Age groups**				
Denmark <30 years	4,379	2,164	4,167	2,546
Denmark 30 years and older	7,043	3,153	11,157	1,103
Finland <30 years	1,631	1,864	5,869	643
Finland 30 years and older	5,959	7,421	17,208	725
Sweden <30 years	9,932	1,412	6,627	1,931
Sweden 30 years and older	10,449	5,159	18,876	1,143
**Deaths**				
Denmark	1,116	470	1,569	109
Finland	820	1,079	2,062	78
Sweden	979	699	2,350	117
**Standardized O/E**				
Denmark	3.8	2.9	2.2	3.0
Finland	3.8	2.4	2.1	3.4
Sweden	2.9	2.8	2.2	3.2
**O/E Circulatory system**				
Denmark	2.2	2.3	1.7	-
Finland	2.7	2.2	1.7	-
Sweden	2.0	2.4	1.6	-
**O/E Cancer**				
Denmark	1.8	1.7	1.7	-
Finland	1.4	1.7	1.5	-
Sweden	1.6	2.0	1.6	-
**O/E Infections**				
Denmark	3.9	5.2	1.9	-
Finland	2.8	2.1	1.9	-
Sweden	2.3	3.5	1.8	-
**O/E Diabetes**				
Denmark	4.0	2.4	2.1	-
Finland	2.5	0.4	0.8	-
Sweden	1.2	3.0	2.1	-
**O/E Other diseases**				
Denmark	7.0	3.5	2.4	-
Finland	6.1	2.6	2.1	-
Sweden	4.0	2.8	1.8	-
**O/E All diseases and medical conditions**				
Denmark	3.6	2.6	1.9	2.4
Finland	3.2	2.2	1.8	3.1
Sweden	2.4	2.4	1.7	2.7
**O/E Suicides**				
Denmark	30.3	38.0	50.5	76.6
Finland	13.4	13.9	26.3	22.2
Sweden	30.1	37.3	46.6	48.2
**O/E Other external causes**				
Denmark	9.9	6.5	3.8	12.3
Finland	14.7	3.2	3.2	4.6
Sweden	8.9	3.8	3.7	3.1
**Life expectancy in risk population. Years**				
Denmark	57.7	64.4	67.8	64.8
Finland	61.9	71.6	71.5	68.1
Sweden	65.0	65.3	69.0	62.6
**Life expectancy in general population. Years**				
Denmark	80.3	80.3	80.3	80.3
Finland	82.5	82.5	82.5	82.5
Sweden	82.6	82.6	82.6	82.6
**Difference in LE. Years**				
Denmark	22.6	15.9	12.5	15.5
Finland	20.6	10.9	11.1	14.5
Sweden	17.6	17.3	13.6	20.0

# The number of cases with personality disorders did not allow detailed analyses of specific cause of death from different medical disorders.

The overall observed/expected ratios for all-cause mortality are increased from 1.7 to 3.6, depending on diagnosis groups and sex, but the figures were highest in substance-related disorders and personality disorders and lowest in affective disorders. The same pattern is reflected when the analysis is restricted to mortality due to all medical conditions and diseases, which was from 1.5 to 3.6 times higher than expected with substance abuse and personality disorders being associated with the highest excess mortality, while affective disorders were associated with lowest excess mortality for both men and women in all three countries.

The observed/expected mortality ratios due to cardiovascular diseases were increased two- to three- fold for both men and women in all diagnostic groups, while figures for infections and other medical causes of death were increased from two- to seven-fold for both men and women. Mortality from cancer was increased from 1.4-fold to 2.0 fold. Regarding diabetes, the mortality figures were generally elevated, but Finnish men and women with schizophrenia and women with affective disorders had reduced mortality associated with diabetes. The total number of deaths with diabetes as the main cause of death was small.

The observed/expected mortality ratios due to other diseases were increased, the risk being from 1.8- to 7-fold. The ratios were highest in patients with substance use disorder and lowest in patients with affective disorders. Detailed analyses in this category showed that mortality due to intestinal tract disorders, especially liver diseases were the most frequently occurring causes of death with observed expected ratios being elevated in all three diagnostic groups in all three countries, and especially high figures were found for substance abuse (data not shown). The second most frequent cause of death in this category was mortality due to substance use disorders, and again these figures were especially high in patients treated for substance abuse (data not shown).

For suicide, the figures were increased from nine- to 37-fold for men and from 13- to 77-fold for women. Affective disorders are associated with the highest risk and substance abuse with the lowest risk for both sexes in all three countries. “Death from other external causes” includes homicides and accidents, of which accidents being the most prevalent; the observed/expected mortality ratios were increased three- to 10-fold for men and three- to 15-fold for women, with patients with substance abuse disorders at the highest risk and patients with affective disorders at the lowest risk.

**Figure 3:**
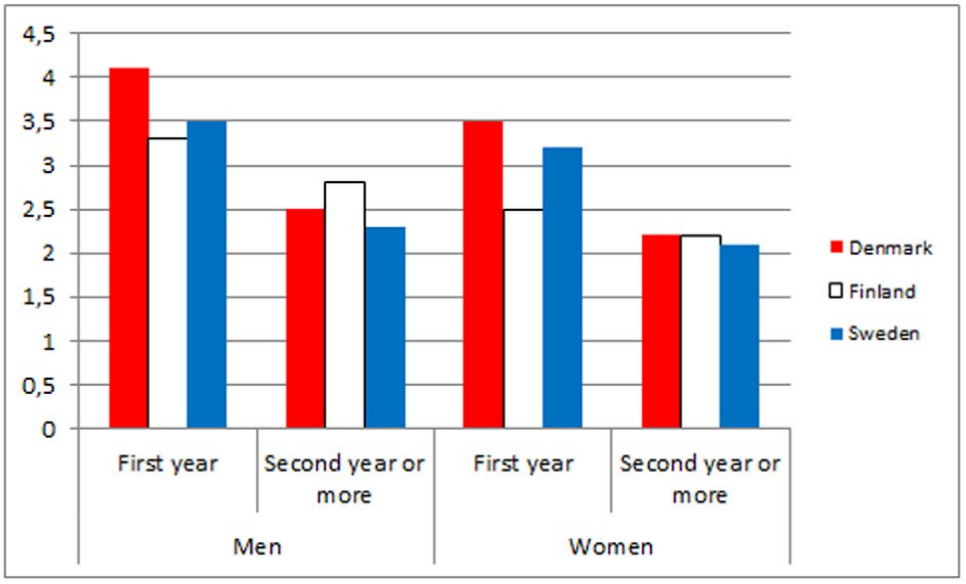
Observed/expected mortality ratios due to medical conditions and diseases during the first years after first discharge versus longer follow-up for men and women in Denmark, Finland and Sweden.

**Figure 4:**
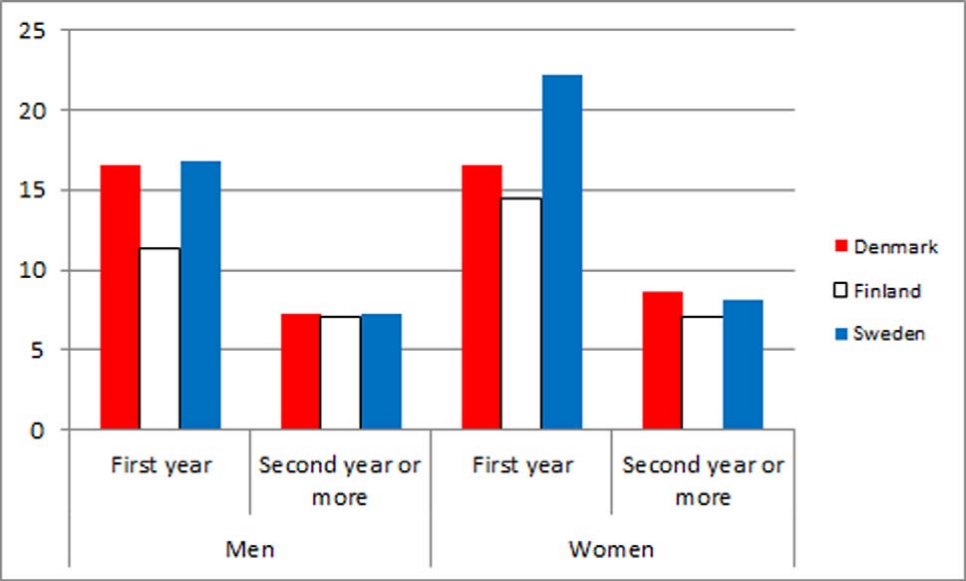
Observed/expected mortality ratios due to external causes of death during the first years after first discharge versus longer follow-up for men and women in Denmark, Finland and Sweden.

In table 3 and figures 3 and 4, the observed/expected mortality ratios during the first years after first discharge compared with longer follow-up are shown for men and women in all three countries. The overall observed/expected mortality ratios due to medical diseases and conditions were clearly higher the first year than in the following years. Regarding suicides and other external causes, the same pattern was even more pronounced.

**Table 3:** Observed/expected mortality during first year vs. second year or more after first discharge due to all diseases and medical conditions and suicides and other external causes in a cohort of patients admitted for psychiatric disorders for the first time in 2000 to 2006 in Denmark, Finland and Sweden.

	Men	Men	Women	Women
	First year	Second year or more	First year	Second year or more
**Number of deaths**				
Denmark	1,704	2,589	1,186	1,857
Finland	2,170	3,740	1,290	2,485
Sweden	1,981	3,296	1,430	2,418
**O/E All diseases and medical conditions**				
Denmark	4.1	2.5	3.5	2.2
Finland	3.3	2.8	2.5	2.2
Sweden	3.5	2.3	3.2	2.1
**O/E suicides and other external causes**				
Denmark	16.6	7.2	16.5	8.6
Finland	11.3	7.1	14.5	7.0
Sweden	16.8	7.3	22.2	8.1

## Discussion

In a study of almost 300,000 patients from Denmark, Finland and Sweden with recent-onset mental disorders, we found overall mortality increased two- to four-fold compared to the general population, and increased cause-specific mortality in all investigated causes of death. The life expectancy was generally approximately 15 years less for women and 20 years less for men compared to the general population.

Different categories of mental disorders ranked as the highest risk for different causes of death, but the general pattern was that substance abuse, schizophrenia and personality disorders were associated with the highest risk for mortality due to diseases and medical conditions; affective disorders and personality disorders were associated with the highest risk of suicide; and substance abuse was associated with the highest risk of death by other external causes, mainly accidents.

Overall, patterns were the same in all three Nordic countries.

The increased mortality due to suicide has been extensively documented in previous publications.[26]–[30] World Health Organization [31] has recommended that suicide prevention in mental disorders should have high priority in Europe, and many countries have developed suicide preventive strategies, including recommendations for special efforts among patients with mental disorders.

Attention should also be drawn to the increased mortality due to other external causes, with accidents being the most important cause. This finding indicates that the patient groups are more prone to accidents e.g. traffic accidents and drug overdoses that are not classified as suicides or deaths with uncertain mode. Perhaps not surprisingly, those with substance abuse disorders constitute the group with the highest risk. As in previous register-based studies in Denmark, Finland and Sweden, we did not include undetermined deaths in the suicide category, as these deaths cannot be classified as certain suicides. However, if some of the deaths classified as undetermined cause of deaths were suicides, the rates reported in this study would be even higher.

Although observed/expected ratios for deaths due to external causes are higher than for all diseases and medical conditions, recent analyses of Danish data show that more years are lost to diseases and medical conditions than to external causes of death for people with bipolar disorder and schizophrenia. Thus, the most of the difference in life expectancy is mainly explained by higher mortality due to diseases and medical conditions. [32] This issue has received less attention; however, the increased mortality and increased risk of metabolic syndrome have been in focus internationally. [33] The European Psychiatric Association (EPA), the European Association for the Study of Diabetes (EASD) and European Society of Cardiology (ESC) have signed a common document with recommendations for monitoring markers of increased risk for metabolic syndrome and cardiovascular diseases. [34].

The excess mortality due to diseases and medical conditions, especially cardiovascular diseases and diabetes, can theoretically be explained by 1) excess morbidity due to unhealthy life style, connected to lack of health literacy and failure of health promotion actions to target this vulnerable population which may have a reduced ability to pick up information on need for behavioral changes [35]–[40] 2) excess iatrogenic morbidity, especially cardiovascular diseases and diabetes, due to adverse effects of psychopharmaceutical medication;[41]–[43] 3) under-diagnosis and under-treatment of physical disorders among mentally ill;[4], [44]–[46] and 4) common genetic risk factors for psychiatric and somatic disorders. [47] These different explanations and their importance need to be further explored in order to plan how to reduce the excess mortality. Deaths with diabetes as the main cause can be due to hyper- or hypoglycaemia, but more often they are related to cardiovascular complications due to diabetes. Deaths in the category other diseases were in many cases related to substance abuse, especially alcohol.

The American Schizophrenia Patient Outcomes Research Team (PORT) recommends weight management for patients with schizophrenia and body mass index higher than 25, [48] but more randomized clinical trials should be carried out to investigate the effectiveness of programmes that aim to improve treatment of medical conditions and facilitate lifestyle changes.

The observed/expected ratios for suicide were consistently lower in Finland than in Denmark and Sweden. As the suicide rate in Finland was almost double compared the rates in Denmark and Sweden during the study period (Eurostat: http://appsso.eurostat.ec.europa.eu/nui/show.do?dataset=hlth_cd_asdr&lang=en, accessed December 28, 2012), it is more likely that the finding reflects the high suicide rates in the general population than low suicide rates among Finnish patients with severe mental disorders. The finding could also be interpreted as a failure to identify and provide hospitalizations for people at risk of suicide in Finland.

Life expectancy for both men and women with substance-related disorders was longer in Sweden than in Denmark and Finland. This is not likely to be explained by a larger proportion of the Swedish population being treated for substance abuse disorders compared to Denmark and Finland. Rather, differences in culture and services for substance-related disorders and legislation making mandatory treatment of substance abuse possible may explain part of this finding. [49] It is known that mortality is higher among people with abuse of illegal drugs than among people with alcohol abuse. Thus, if there are proportionally more people with abuse of illegal drugs than people with alcohol abuse in Denmark than in Sweden, then also this could explain the difference between Denmark and Sweden. However, it would not explain the difference between Finland and Sweden, as comparatively, rather few people have had abuse of illegal drugs in Finland. These considerations are supported by the number of drug related deaths per 100.000 inhabitants in the three countries, which in 2009 was 4.0 in Denmark, 2.9 in Finland and 2.83 in Sweden. (European Monitoring Centre for Drugs and Drug addiction: http://www.emcdda.europa.eu/publications/country-overviews, Denmark, Finland and Sweden accessed December 28, 2012).

Our data indicate that excess mortality, irrespective of whether it is due to diseases or to external causes, peaks during the first year after discharge. This has previously been documented for suicide in several studies [16], [30], [50], [51] Regarding deaths due to diseases and medical conditions, excess death following hospitalization due to a mental disorder not only indicates that a current mental disorder is a risk factor contributing to death from a physical disorder; it also indicates a systematic failure of the health system to prevent, identify and treat physical diseases during hospitalization for a mental disorder. This interpretation is supported by studies documenting that patients with psychiatric disorders receive less treatment for physical conditions than people without psychiatric disorders.[44], [52]–[54] Theoretically, the finding could be explained by people being treated for diseases and medical conditions in health services are more likely to be diagnosed with psychiatric disorder and admitted at psychiatric department (Berkson’s bias).

The data presented can provide a sound basis for action to reduce the excess mortality, and it indicates the necessity to address mortality due to both diseases and medical conditions *and* external causes for injuries and poisoning, also including suicide.

### Strengths and Limitations

The strengths of the study are that it represents a very large population-based register data and that the analyses were conducted on total patient populations in three countries with similar results. This indicates the robustness of the findings on this important public health issue. Compared to other studies, this study is based on a larger population. The study is based on a recent cohort, and therefore the results are still valid for current psychiatric practice.

The study population only included persons who at least once during the period 2000 to 2006 had an inpatient care episode. Thus, it must be assumed that the study population represents the most severely ill patients with mental disorders, while patients treated in outpatient psychiatric services or only in primary care were not included.

Co-morbidity was not analyzed, and it is possible that the high mortality figures in schizophrenia, affective disorders and personality disorders are partly explained by co-morbid substance abuse.

Although we included total national data, there were too few cases for some of the detailed analyses.

The study is based on rather crude register-based data from three countries, and the data cannot serve as the basis for more detailed analyses of the importance of each of the hypothesized mechanisms behind the excess mortality. We lack information about life style, medication and treatment for medical diseases. We are therefore unable to disentangle the relative importance of each of the mechanisms.

Patients with mental retardation (F70–F79) were excluded as several of the disorders are associated with increased mortality, but their number was low. These diagnoses were included in our comparison data, as it included total populations in all the three study countries. If we had been able to exclude people with learning disabilities – and with mental disorders – from the general population sample, the differences between the cases and the comparison group would have been larger.

### Conclusions

Our study provides further and conclusive evidence that there is alarming increased mortality due to both diseases and medical conditions and external causes, and that life expectancy is dramatically reduced in patients with severe mental disorders across a wide range of diagnostic categories. These figures call for actions to prevent the high mortality. Further research should disentangle the role of adverse effects of medication, poor life style, and lack of diagnosis and treatment of medical conditions. Randomized clinical trials should be carried out to explore the effect of lifestyle interventions and interventions aiming to reduce the treatment gap with regard to medical diseases.
